# Characterization of GH2 and GH42 β-galactosidases derived from bifidobacterial infant isolates

**DOI:** 10.1186/s13568-019-0735-3

**Published:** 2019-01-19

**Authors:** Valentina Ambrogi, Francesca Bottacini, Joyce O’Sullivan, Mary O’Connell Motherway, Cao Linqiu, Barry Schoemaker, Margriet Schoterman, Douwe van Sinderen

**Affiliations:** 1 APC Microbiome Ireland, University College Cork, Western Road, Cork, Ireland; 2 School of Microbiology, National University of Ireland, Western Road, Cork, Ireland; 3 FrieslandCampina, Amersfoort, The Netherlands

**Keywords:** β-Galactosidases, Bifidobacteria, Lactose, HMOs, Infant gut microbiota

## Abstract

Bifidobacteria are among the first and most abundant bacterial colonizers of the gastrointestinal tract of (breast-fed) healthy infants. Their success of colonising the infant gut is believed to be, at least partly, due to their ability to metabolize available carbon sources by means of secreted or intracellular glycosyl hydrolases (GHs). Among these, β-galactosidases are particularly relevant as they allow bifidobacteria to grow on β-galactosyl-linked saccharidic substrates, which are present in copious amounts in the milk-based diet of their infant host (e.g. lactose and human milk oligosaccharides). In the present study we employed an in silico analysis to identify GH family 2 and 42 β-galactosidases encoded by typical infant-associated bifidobacteria. Comparative genome analysis followed by characterisation of selected β-galactosidases revealed how these GH2 and GH42 members are distributed among these infant-associated bifidobacteria, while their hydrolytic activity towards growth substrates commonly available in the infant gut were also assessed.

**Electronic supplementary material:**

The online version of this article (10.1186/s13568-019-0735-3) contains supplementary material, which is available to authorized users.

## Introduction

Bifidobacteria are common members of the human gut microbiota, and are especially abundant in the gastrointestinal tract (GIT) of healthy, breast-fed infants (Milani et al. 2017). The presence of bifidobacteria in the gut is believed to provide a number of health benefits for the human host, although the mechanisms by which such benefits are delivered are currently not fully understood (Arboleya et al. 2016; Milani et al. 2017). Bifidobacteria are saccharolytic microorganisms and their ability to utilize complex dietary glycans or host-derived mucins is an important property to assist in their establishment and persistence into the GIT (Koropatkin et al. 2012; Milani et al. 2015; Riviere et al. 2016). The saccharolytic metabolism of bifidobacteria is facilitated by a large array of carbohydrate degrading enzymes, in particular glycosyl hydrolases (GHs), which provide the capacity to directly or indirectly (through syntropy) utilize a range of glycan substrates available in the gut (Milani et al. 2015; Turroni et al. 2018a). Bifidobacteria commonly represent the dominant component of the gut microbiota of healthy, breast-fed infants (Roger et al. 2010), a phenomenon that is believed to be due in part by their ability to metabolize human milk oligosaccharides (HMOs), which contain one or more β-linked galactose moieties (Fuhrer et al. 2010). Among the glycosyl hydrolases encoded by bifidobacteria (Milani et al. 2015) β-galactosidases (which are members of GH families 2 and 42) (van den Broek et al. 2008) have been described to participate to the utilization of (human) milk and milk-based substrates (i.e. lactose, HMOs as well as GOS) in *Bifidobacterium bifidum*, *Bifidobacterium longum* subsp. *infantis* and *Bifidobacterium breve* (Garrido et al. 2013; Goulas et al. 2007; James et al. 2016; Miwa et al. 2010; Moller et al. 2001; O’Connell Motherway et al. 2013; Yoshida et al. 2012). In addition, β-galactosidases in *B. bifidum* can also participate to the degradation of mucin (Turroni et al. 2010), while in *B. breve* such enzymes are involved in the degradation of the plant polymer galactan (O’Connell Motherway et al. 2011).

Human milk glycans can be quite diverse and they are composed of 13 core structures generated through the elongation of lactose at the reducing end with one or more β1,3-linked lacto-*N*-biose (type-I chain) and/or β1,3/6-linked *N*-acetyllactosamine units (type-II chain) (Urashima et al. 2012). These core structures (including lactose itself) can in turn be substituted at terminal positions by fucose connected via α1,2/3/4 links, and/or sialic acid residues attached by α2,3/6 links (Smilowitz et al. 2014). Notably, HMOs are especially rich in type-I chains and constitute a characteristic feature of human milk (Urashima et al. 2012).

Some of the glycosidic linkages and monosaccharides found in HMOs are also present in mucins found in the intestine, for example the presence of β-linked galactose and *N*-acetyl-glucosamine, as well as fucose and sialic acid residues which are substituted to mucin via α1,2/3/4 and α2,3/6 linkages (Tailford et al. 2015).

Recent genome analyses have highlighted metabolic pathways responsible for the degradation of HMOs and mucin-type *O*-glycans in certain infant-derived bifidobacteria. With regards to HMO utilization it has been shown that *B. bifidum* and *B. longum* subsp. *infantis* can utilize many different HMOs directly (Asakuma et al. 2011; Garrido et al. 2016; Sela 2011; Ward et al. 2007). In contrast, *B. breve* and *B. longum* subsp. *longum* can only metabolize a small number of these directly, thus relying on cross-feeding strategies (Asakuma et al. 2011; Egan et al. 2014; LoCascio et al. 2007; Ward et al. 2007). *B. bifidum* is the only bifidobacterial species described so far to be able to utilize mucin-type *O*-glycans (Turroni et al. 2010), and the products of (extracellular) mucin degradation constitute growth substrates for other (bifido)bacterial species, revealing that bifidobacteria can adopt a syntrophic strategy to access different substrates available in the gut (Egan et al. 2014; Turroni et al. 2018b).

The complex structural and functional heterogeneity of HMOs in mother’s milk is presumed to be essential in conferring the bifidogenic activity and associated health benefits to the host, yet it also represents the main limitation for the deliberate incorporation of HMOs in infant formulations (Akkerman et al. 2018). For this reason alternative ways of recreating the beneficial effects of human milk have focused on the enzymatic production of other non-digestible, lactose-derived prebiotics, such as GOS produced by β-galactosidases that have transgalactosylation activities (Macfarlane et al. 2008).

Based on the above, the relevance of diet-based, lactose-derived oligosaccharides (e.g. HMOs and GOS) in supporting bifidobacterial establishment and persistence in the gut is obvious, especially during early life when bifidobacteria are particularly abundant (Milani et al. 2017). The current study focused on a survey and subsequent hydrolytic characterization of (selected) β-galactosidases identified from sequenced strains of bifidobacterial species which have been previously shown to be members of the infant gut microbiota (i.e. *B. breve*, *B. bifidum*, *B. longum* subsp. *longum* and *B. longum* subsp. *infantis*) (Lewis and Mills 2017; Milani et al. 2017; Turroni et al. 2012). Following comparative genome analysis, our analysis focused on a number of selected β-galactosidases of which hydrolytic capabilities were assessed for various β-galactoside-containing substrates.

## Materials and methods

### In silico genome analysis to identify putative β-galactosidase-encoding genes

For comparative purposes, genomic datasets, consisting of 34 genome sequences (Table 1) from four representative infant-derived bifidobacterial (sub)species (i.e. *B. breve*, *B. bifidum*, *B. longum* subsp. *longum* and *B. longum* subsp. *infantis*) were retrieved from GenBank (https://www.ncbi.nlm.nih.gov/genbank). Of the corresponding 34 bifidobacterial strains, we selected six strains as representative reference strains for our analysis as their genome had been sequenced to completion and because these strains are available within our culture collection [i.e. *B. breve* UCC2003 (GenBank: CP000303), *B. breve* JCM 7017 (GenBank: CP006712), *B. breve* JCM 7019 (GenBank: CP006713), *B. bifidum* LMG 13195 (WGS: AMPL01000000), *B. longum* subsp. *longum* NCIMB 8809 (GenBank: CP011964) and *B. longum* subsp. *infantis* ATCC 15697 (GenBank: CP001095)]. The selected genomes were searched for predicted β-galactosidase-encoding genes on the basis of a functional annotation supported by PFAM analysis (http://pfam.xfam.org/), combined with information retrieved from the Cazy database (http://www.cazy.org). Predictions were refined using comparative genome analysis with detection of orthologous β-galactosidase-encoding genes across genomes using BLASTP searches (Altschul et al. 1990). BLASTP alignments were performed using a stringency of 50% of identity across 50% of the length of the examined proteins, with a cut-off E-value of < 0.0001. Predicted β-galactosidase-encoding genes were then grouped in clusters of orthologs using the cd-hit pipeline (http://weizhongli-lab.org/cd-hit/) with a clustering threshold of 70% identity across 90% of the corresponding protein length (Bottacini et al. 2014).

**Table 1:** Bifidobacterial strains used for comparative analyses

Species	Strain	Accession number WGS
*B. bifidum*	JCM 1254	BBBT00000000
*B. bifidum*	DSM 20215	–
*B. bifidum*	DSM 20456	JDUM00000000
*B. bifidum*	NCIMB 41171	AKCA01000000
*B. bifidum*	PRL2010	CP001840
*B. bifidum*	S17	CP002220
*B. bifidum*	BGN4	CP001361
*B. bifidum*	IPLA20015	AMPM01000000
*B. bifidum*	LMG 11041	JGYO01000000
*B. bifidum*	LMG 13195	AMPL01000000
*B. breve*	2L	AWUG00000000
*B. breve*	JCM 7019	CP006713
*B. breve*	UCC2003	CP000303
*B. breve*	31L	AWUF01000000
*B. breve*	NCFB 2258	CP006714
*B. breve*	S27	CP006716
*B. breve*	12L	CP006711
*B. breve*	JCM 7017	CP006712
*B. breve*	JCP 7499	AWSX01000000
*B. longum* subsp. *infantis*	ATCC 15697	CP001095
*B. longum* subsp. *infantis*	EK3	JNWB01000000
*B. longum* subsp. *infantis*	157F	AP010890
*B. longum* subsp. *infantis*	CCUG 52486	ABQQ01000000
*B. longum* subsp. *longum*	NCIMB 8809	CP011964
*B. longum* subsp. *longum*	EK13	JNWD01000000
*B. longum* subsp. *longum*	F8	FP929034
*B. longum* subsp. *longum*	KACC 91563	CP002794
*B. longum* subsp. *longum*	VMKB44	JRWN01000000
*B. longum* subsp. *longum*	ATCC 55813	ACHI01000000
*B. longum* subsp. *longum*	BBMN68	CP002286
*B. longum* subsp. *longum*	JCM 1217	AP010888
*B. longum* subsp. *longum*	LMG 13197	JGYZ01000000
*B. longum* subsp. *longum*	JDM301	CP002010
*B. longum* subsp. *longum*	CECT 7347	CALH01000000

Phylogenetic inference was conducted using the MEGA7 suite (http://www.megasoftware.net/). Protein sequence alignments were performed using the Muscle module available within MEGA7 and the resulting phylogenetic tree was built using the neighbour joining approach with statistical assessment based on 1000 bootstrap replicates.

### Growth conditions and strain manipulation

Bifidobacterial strains were cultured in de Man Rogosa and Sharpe (MRS) medium supplemented with cysteine-HCl (0.05% w/v) and incubated at 37 °C degrees under anaerobic conditions using an anaerobic chamber (Davidson and Hardy, Belfast, Ireland). *L. lactis* strain NZ9000 (University of Groningen, The Netherlands) was selected for cloning (de Ruyter et al. 1996), overproduction and purification purposes as several bifidobacterial GH-encoding genes had previously been successfully cloned and expressed in this host (James et al. 2016; O’Connell et al. 2013; O’Connell Motherway et al. 2013). *L. lactis* NZ9000 was grown in M17 broth (Oxoid, UK) supplemented with 0.5% glucose (GM17) at 30 °C degrees (Terzaghi and Sandine 1975). *L. lactis* NZ9000 cells carrying (suspected) recombinant plasmids were selected on GM17 agar containing chloramphenicol (Cm 5 µg ml^−1^), and supplemented with X-gal (5-bromo-4-chloro-3-indolyl-ß-d-galactopyranoside) (40 µg ml^−1^) to visually identify recombinant clones expressing β-galactosidase activity.

Chromosomal DNA was isolated from *B. breve* UCC2003, *B. bifidum* LMG 13195, *B. longum* subsp. *longum* NCIMB 8809 and *B. longum* subsp. *infantis* ATCC 15697 as previously described (O’Riordan and Fitzgerald 1998). Plasmid extraction from *L. lactis* was achieved using the Roche High Pure Plasmid Isolation Kit (Roche Diagnostics GmbH, Mannheim, Germany). For *L. lactis* an initial lysis step was included where cells are incubated in lysis buffer containing 30 mg ml^−1^ of lysozyme for 30 min at 37 °C. Oligonucleotide PCR primers used in the study were synthesized by Eurofins (Ebersberg, Germany; Table 2). Standard PCRs were performed using Thermo Scientific Extensor Hi-Fidelity PCR Master mix (Thermo Scientific), while high fidelity PCR was achieved using Q5 High-Fidelity DNA Polymerase (New England BioLabs, New Brunswick). PCR fragments were purified with Roche High Pure PCR Product Purification Kit (Roche Diagnostics GmbH). Plasmid pNZ8150 was used as cloning and nisin-inducible expression vector. DNA fragments encompassing predicted β-galactosidase-encoding genes and the pNZ8150 vector were restricted with specific restriction enzymes (Table 2) according to the supplier’s instructions (Roche Diagnostics, East Sussex, United Kingdom). Ligation was achieved with T4 DNA ligase according to the manufacturer’s instructions (Promega, Wisconsin, USA, worldwide.promega.com). Electroporation of (ligated) plasmid DNA into *L. lactis* was performed as described previously (Wells et al. 1993).

**Table 2:** Oligonucleotide primers used in this study

Gene	Primer	Sequence	Restriction enzymes
Bbr_0010	Fw	tgcatcGATAT**C**catcaccatcaccatcaccatcaccatcacatgcatcaccatcaccatcaccatcacc	*Eco*RV
	Rv	tgcgcaTCTAGAtcagatgagttcgagtgtcac	*XBa*I
Bbr_0285	Fw	tgcatcGATATCatgcatcaccatcaccatcaccatcaccatcacatggagcgaatccaatacccc	*Eco*RV
	Rv	tgcgcaTCTAGAtcacacctgcacgtagccg	*XBa*I
Bbr_0310	Fw	tgcatcGATATCcatcaccatcaccatcaccatcaccatcacatggggacgacaggacacagc	*Eco*RV
	Rv	tgcgcaTCTAGAtcaactcttttcgattgcg	*XBa*I
Bbr_0420	Fw	tgcatcCCCGGGcatcaccatcaccatcaccatcaccatcacatgactactcgtagagc	*Xma*I
	Rv	ctcgaaTCTAGActagcaggacgttttagcg	*XBa*I
Bbr_0529	Fw	tgcatcGATATCcatcaccatcaccatcaccatcaccatcacatggaacatcgcgaattcaag	*Eco*RV
	Rv	tgcgcaTCTAGAttacagctttaccaccagcac	*XBa*I
Bbr_1552	Fw	tgcatcGATATCcatcaccatcaccatcaccatcaccatcacatgaacacaaccgacgatcag	*Eco*RV
	Rv	tgcgcaTCTAGAtcagatgagttcgaggttcac	*XBa*I
Bbr_1689-1690	Fw	tgcatcCAGCTGcatcaccatcaccatcaccatcaccatcacatgagcaagcagaacgattg	*Pvu*II
	Rv	tgcgcaTCTAGAgctggcatcttcctgaacg	*XBa*I
B7017_2031	Fw	tgcatcGAATTCcatcaccatcaccatcaccatcaccatcacatgaccgacaccatggcacacacacaacc	*Eco*RI
	Rv	tgcatcTCTAGActgatgatgaaggatgactgaagccg	*XBa*I
B216_06500	Fw	tgcatc ATTTAAAT atg catcaccatcaccatcaccatcaccatcac gtcaataccgttagggttgt	*Swa*I
	Rv	tgcatc TCTAGAcccggggagactcgcgagagt	*XBa*I
B216_08266	Fw	tgcatcATTTAAATcatcaccatcaccatcaccatcaccatcacatgagtaaacgcagaaagcacag	*Swa*I
	Rv	tgcgcaTCTAGAgtatgtcgcgtgtcaccg	*XBa*I
B216_08730	Fw	tgcatcGATATCatgcatcaccatcaccatcaccatcaccatcacgtgcgcgcgcgacgtgactttg	*Eco*RV
	Rv	tgcatc TCTAGA aacgttgaaatagagccggaaac	*XBa*I
B216_09411	Fw	tgcatc ATTTAAATatg catcaccatcaccatcaccatcaccatcac ttcattccccggtactacg	*Swa*I
	Rv	tgcatc TCTAGA atccgatacccgtacccgtg	*XBa*I
B216_09623	Fw	tgcatcATTTAAATcatcaccatcaccatcaccatcaccatcacatgaacacaaccgacgatcagc	*Swa*I
	Rv	tgcgcaTCTAGAatgagcgagaggacctggcg	*XBa*I
B8809_0321	Fw	tgcatcATTTAAATcatcaccatcaccatcaccatcaccatcacatgactactcatagagcatttag	*Swa*I
	Rv	tgcatc TCTAGAcattctagcgcggtttag	*XBa*I
B8809_0415	Fw	tgcatcGATATCcatcaccatcaccatcaccatcaccatcacatggaacgtaaagagttcaagtgg	*Eco*RV
	Rv	tgcatcTCTAGAccgttgggtaattaggcgct	*XBa*I
B8809_0611	Fw	tgcatcGATATCcatcaccatcaccatcaccatcaccatcacatgacagacgtcacacatgtcg	*Eco*RV
	Rv	tgcatc TCTAGAtgcacggtggactatcggatc	*XBa*I
B8809_1361	Fw	tgcatcGATATCcatcaccatcaccatcaccatcaccatcacatgcagcatcccatccccaccac	*Eco*RV
	Rv	tgcatc TCTAGAcagcacgataaagaagctccctcg	*XBa*I
Blon_2016	Fw	tgcatcGATATCcatcaccatcaccatcaccatcaccatcacatggaacatagagcgttcaagt	*Eco*RV
	Rv	tgcatcTCTAGAcggctccctgctgcgatga	*XBa*I
Blon_2123	Fw	tgcatcGATATCcatcaccatcaccatcaccatcaccatcacatggtgcgtgcgcgacgtgactt	*Eco*RV
	Rv	tgcatcTCTAGAaccatgtacgtcggcaccgt	*XBa*I
Blon_2416	Fw	tgcatcGATATCcatcaccatcaccatcaccatcaccatcacatgaccgacaccatggcaca	*Eco*RV
	Rv	tgcatcTCTAGAcggttgctgacttgggatat	*XBa*I

### Protein overproduction and purification

For bifidobacterial protein overproduction 0.4 ml of M17 broth supplemented with 0.5% glucose was inoculated with a 2% inoculum of *L. lactis* strain harbouring the pNZ8150 cloning vector containing a (predicted) β-galactosidase-encoding gene, and cultivated at 30 °C until the culture reached an Optical Density (OD_600 nm_) of 0.5. At this point targeted protein expression was induced by the addition of filter sterilised cell free supernatant of a nisin-producing strain *L. lactis* NZ9700 (0.2% v/v) (de Ruyter et al. 1996) and incubation was continued at 30 °C for 90 min. Cells were harvested by centrifugation (5000 rpm for 10 min), the obtained pellet was recovered in lysis buffer (50 mM sodium phosphate buffer, pH 8; 300 mM NaCl; 10 mM Imidazole) and cells were disrupted by bead beating (1 min, three times). Cell debris was removed by centrifugation (15,000 rpm for 20 min at 4 °C) and the resulting supernatant, representing the crude cell extract, was used for (His-tagged) protein purification using a nickel-nitrilotriacetic acid column (Qiagen GmbH) according to the manufacturer’s instructions (QIAexpressionist, June 2003). Elution fractions were analysed by SDS-polyacrylamide gel electrophoresis (SDS-PAGE), as described previously (Laemmli 1970), on a 12.5% polyacrylamide (PAA) gel. After electrophoresis, PAA gels were fixed and stained with Coomassie brilliant blue to identify fractions containing the purified protein. For molecular weight estimation of (purified) proteins the Prestained Protein Marker, Broad Range (7–175 kDa) was used (New England BioLabs, Hertfordshire, United Kingdom).

### Beta-galactosidase assay: crude cell extract

Colorimetric and spectrophotometric assay was performed to evaluate the hydrolytic activity of predicted β-galactosidases towards the artificial substrate *O*-nitrophenyl β-d-galactopyranose (ONPG assay). This assay was performed at 30 °C according to a previously published protocol (Miller, Cold Spring Harbor, 1972). Following the development of a yellow color the reaction was terminated by the addition of 250 µl 1 M sodium carbonate. This was followed by measurement of absorbance at a wave length of 420 nm. Calculation of β-galactosidase activity was performed according to the following formula: β-gal unit = 1000*OD_420 nm_/Time*V*OD_600 nm_, with incubation time of 5 min (or longer if required) and volume of resuspended pellet (V) of 0.05 ml.

### Hydrolysis assay and product analysis

Hydrolysis assays of β-galactosidase activity were performed on the following commercially available substrates: lactose, lactulose, two different galactotrioses (Galβ1-4Galβ1-4Gal and Galα1-3Galβ1-4Gal), two different galactobioses (Galβ1-6Gal and Galβ1-4Gal), lacto-*N*-tetraose (LNT), lacto-*N*-neotetraose (LNnT), 2′-fucosyllactose (2′-FL) and 3-fucosyllactose (3-FL). The hydrolysis assays were performed using the following conditions: 250 µl enzyme solution was incubated with 0.1 mg ml^−1^ of a particular carbohydrate in MOPS [3-(*N*-morpholino) propanesulfonic acid] buffer pH 6.5 in a total volume of 1 ml at 37 °C. Aliquots of 250 μl were collected after 1, 3 and 24 h from the start of the reaction and following collection samples were immediately heated at 80 °C for 5 min in order to terminate the reaction, after which they stored at − 20 °C until analysis by high-performance anion-exchange chromatography with pulsed amperometric detection (HPAEC-PAD) employing a CarboPac PA1 (Thermo Scientific) analytical-anion exchange column (dimensions, 250 mm by 4 mm) with a CarboPac PA1 (Thermo Scientific) guard column (dimensions, 50 mm by 4 mm) and a detector (ED40) in the pulsed amperometric detection PAD mode (Dionex, Thermo Scientific). Elution was performed at a constant flow rate of 1.0 ml min^−1^ at 30 °C using the following eluents for the analysis: (A) 200 mmol NaOH, (B) 100 mmol NaOH, 550 mmol sodium acetate (NaAC), and (C) Milli-Q water. The elution gradient is reported in Table 3. The obtained data sets were then analysed employing CHROMELEON software Ver.6.70 (Dionex, Thermo Scientific).

**Table 3:** Elution gradient used in HPAEC-PAD

Minute	Concentration NaOH (mmol l^−1^)	Concentration NaAC (mmol l^−1^)
0–50	100	0
50–51	0	0
51–56	0	16
56–61	0	100

## Results

### Comparative analysis of β-galactosidase-encoding genes

In order to identify putative β-galactosidase-encoding genes of infant-associated bifidobacterial strains, we initially selected the genomes of six strains to act as representatives for four bifidobacterial (sub) species (see “Materials and methods” section) that are typically prevalent and abundant in the (healthy) infant gut microbiota.

Putative β-galactosidase-encoding genes were identified on the genome sequence of each of these six reference strains (representing four species) based on manual searches and cross-validation of the obtained result with PFAM searches and Cazy database (see “Materials and methods”). The obtained list of predicted β-galactosidase-encoding genes was then used for further comparative analyses, aimed at identifying homologous genes across a more extensive set of 34 bifidobacterial genomes, belonging to the same (sub)species as the reference set (Table 1). Employing this comparative search approach we established that the total number of predicted GH2 and GH42 β-galactosidase-encoding genes encoded by (34 representatives of) these four bifidobacterial species is 137, of which 44, 45 and 48 were assigned to *B. breve*, *B. bifidum* and *B. longum* subsp. *longum* spp., respectively (Additional file 1: Table S1).

### Comparative sequence analysis and phylogeny of predicted β-galactosidase genes

**Fig. 1:**
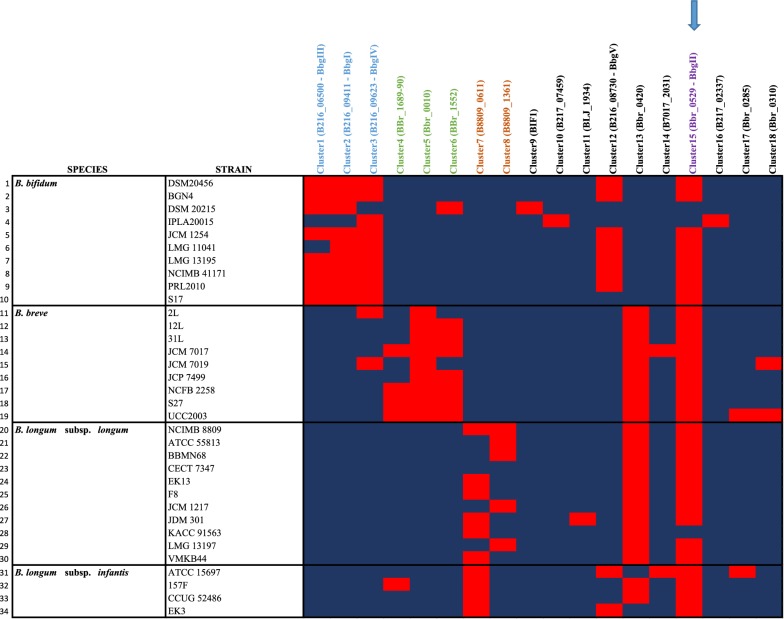
Comparative analysis of β-galactosidases in infant-derived bifidobacterial (sub)species. Heatmap showing presence (red) and absence (blue) of 18 clusters of β-galactosidase-encoding genes across the 34 bifidobacterial representatives of *B. breve*, *B. bifidum*, *B. longum* subsp. *longum* and *B. longum* subsp. *infantis*. A blue arrow highlights Cluster 15, which is present across members of all the four (sub)species, while blue, green and brown colours identify clusters observed only in *B. bifidum*, B*. breve* and *B. longum* species, respectively

To establish if a putative β-galactosidase-encoding gene is represented by homologs across other strains or (sub)species, we organized the 137 identified β-galactosidases in 18 orthologous families (here referred as clusters based on a cut-off value of 70% of similarity over 90% of sequence length; see “Materials and methods”) (Additional file 1: Figure S1). This analysis revealed that members of each of the 18 orthologous clusters are variably distributed across a given species, where certain β-galactosidases seem to be more species-specific (e.g. Clusters 1–3 for *B. bifidum*, Clusters 4–6 for *B. breve* and Clusters 7–8 for *B. longum* subsp. *longum*), while others are present among all members of the four (sub)species (e.g. Cluster 15) (Fig. 1 and Additional file 1: Table S1).

**Fig. 2:**
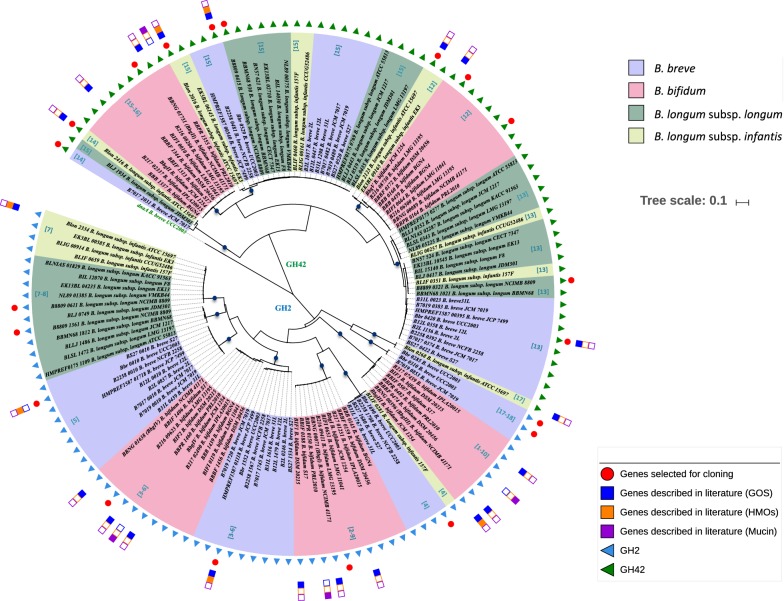
Phylogenomics of β-galactosidases. Neighbour-joining tree based on the alignment of 137 putative β-galactosidases identified across 34 members of infant-derived bifidobacterial (sub)species. A red circle highlights the genes used for experimental assessment in this study, while blue and orange squares identify those genes of which involvement in the assimilation of GOS and HMOs have been reported in literature. Circles on the phylogenetic tree highlight nodes with bootstrap values above 90% and dark blue circles identify the 14 phylogenetic groups defined by our analysis

As mentioned above comparative sequence analysis organized the 137 identified β-galactosidases into 18 clusters of orthology (Fig. 1). A subsequent analysis was carried out in order to establish the phylogenetic relationships between bifidobacterial β-galactosidases. This analysis resulted in the generation of a phylogenetic tree which subdivided these 137 genes into two distinct clades, corresponding to members that either belong to GH family 2 or GH family 42 (Fig. 2). Within these GH2 and GH42 clades, our analysis revealed that the β-galactosidase-encoding genes are further distributed across 14 distinct phylogenetic groups (or subclades defined by nodes of bootstrap value above 90%), where genes from strains belonging to the same species often cluster together (Fig. 2).

### Cloning of bifidobacterial β-galactosidase-encoding genes

**Fig. 3:**
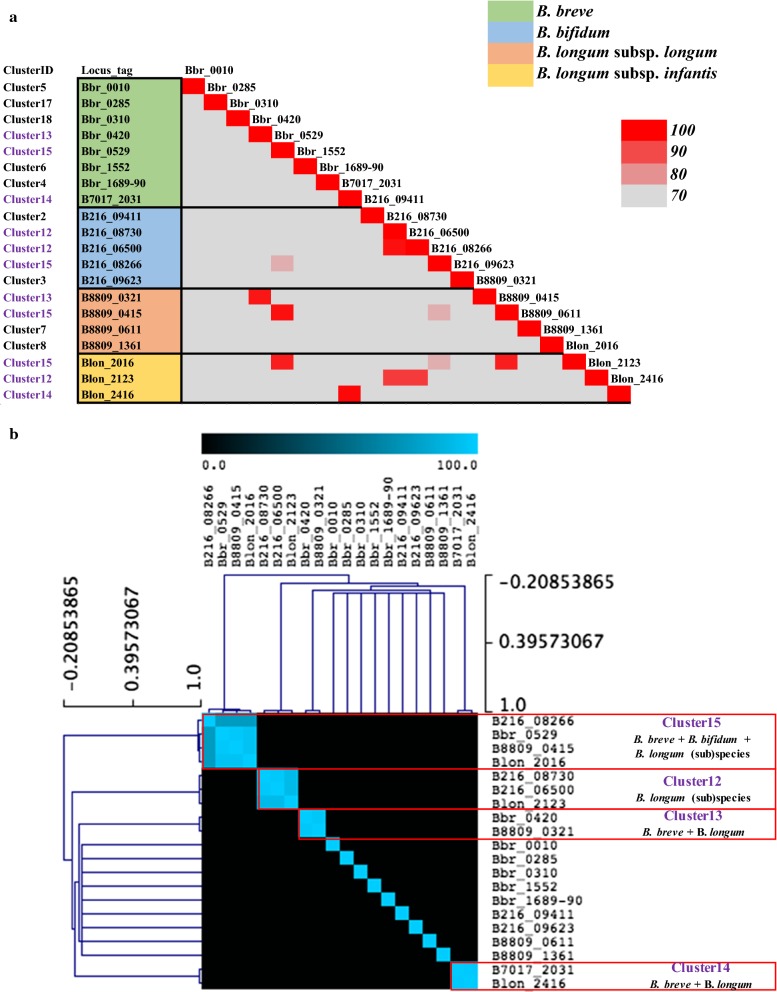
Distribution of β-galactosidases selected for functional assessment across the five bifidobacterial reference strains. **a** Similarity plot showing the percentage of similarity of the 20 selected β-galactosidases across the reference strains (cut-off of 70% of identity over 90% of protein length). For each gene the relative cluster of appartenance from comparative genomics is also indicated. **b** Hierarchical clustering analysis showing the co-occurrence of the 20 selected β-galactosidases and their cluster of orthology across the reference genomes and bifidobacterial (sub)species

Based on the outcome of the phylogenetic analyses, a total of 20 most diverse β-galactosidases (present in the six representative genomes available within our culture collection, while also considering practical limitations of cloning this large number of genes) were selected as candidates for further experimental validation, which in most cases locate in separate branches of the phylogenetic tree (Fig. 3a). This selection therefore captures much of the observed diversity among the identified (putative) β-galactosidases (Fig. 3). Notably, of these 20 selected β-galactosidases, four (corresponding to locus tags B216_08266, Bbr_0529, B8809_0415 and Blon_2016) are orthologous genes within Cluster 15, of which members are present across all four infant-derived bifidobacterial (sub)species, while the remaining 16 selected genes are present only within certain (sub)species (Fig. 3b).

In order to investigate if the 20 putative β-galactosidase-encoding genes as selected above do in fact encode this enzymatic activity, cloning and (over)expression of each of these bifidobacterial genes in *Lactococcus lactis* was performed. The 20 β-galactosidase-encoding genes were therefore amplified by PCR using primer pairs, one of which included a six His-tag-encoding sequence (to aid in subsequent purification), and cloned into a nisin-inducible expression vector in *L. lactis* (see “Materials and methods”). Two out of the 20 predicted β-galactosidase-encoding genes (corresponding to locus tags B216_06500, B216_09411) appeared to be unclonable in *L. lactis* (as based on multiple failed attempts). One explanation could be the plasmid instability due to the (size of the) inserted gene, or toxicity of their products in the cloning host. In one case (i.e. Blon_2123 from *B. longum* subsp. *infantis* ATCC 15697), we found that the cloned gene contained a stop codon located 100 bp downstream of the start of the gene, thus making this a pseudogene. It is worth mentioning that BLAST analysis of this particular sequence against the deposited genome sequence of *B. longum* subsp*. infantis* ATCC 15697 showed that the publicly available sequence does not contain a stop codon at this point, suggesting that this mutation may be unique to our stock of *B. longum* ATCC 15697 (NB. the chromosomal sequence of this stock was verified by sequence analysis of a PCR product that encompassed the relevant section of Blon_2123). The obtained *L. lactis* transformants (or at least a proportion of the colonies corresponding to a given transformation) from the remaining 17 cloning efforts were in 14 cases shown to form easily identifiable blue colonies on plates including the chromogenic substrate X-gal (yet in the absence of the inducer nisin) (Table 4). This result clearly indicates that the majority of the cloned genes indeed encodes a β-galactosidases active on this artificial substrate. In three cases (i.e. corresponding to the cloning of genes with locus tags Bbr_0285, Bbr_0310 and B7017_2031) transformants only formed white colonies (though they contained the expected recombinant plasmid as verified by sequencing), suggesting that either the encoded product does not encode a β-galactosidase capable of cleaving X-Gal, or that the cloned gene was not (highly) expressed or its product not properly folded (Table 4). The plasmid content of a selection of positively identified clones for each cloning was sent for sequencing to validate sequence integrity of the cloned gene. In summary, of the total of 20 selected (putative) β-galactosidase-encoding genes, 17 were successfully cloned (as an intact gene), of which 14 turned out to encode β-galactosidase activity as based on the applied X-gal plate assay.

**Table 4:**
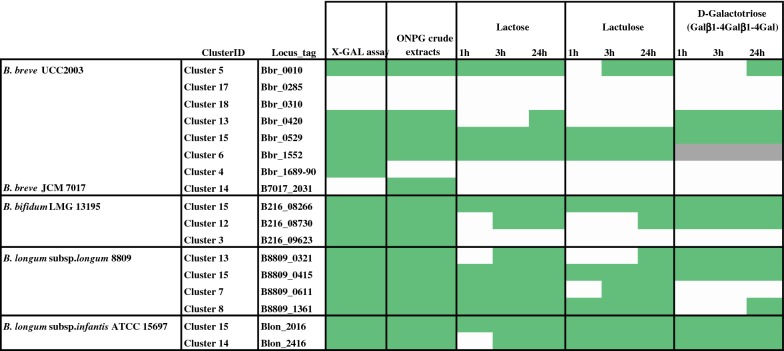
Hydrolysis performance of the 17 cloned β-galactosidases tested on substrates lactose, lactulose and d-galactotriose

Key to colour coding: Substrate hydrolysis observed (green), no hydrolysis (white) and substrate not assayed (grey). Results of X-gal and ONPG assays are also reported for comparative purposes

### β-galactosidase assays: crude cell extract

**Fig. 4:**
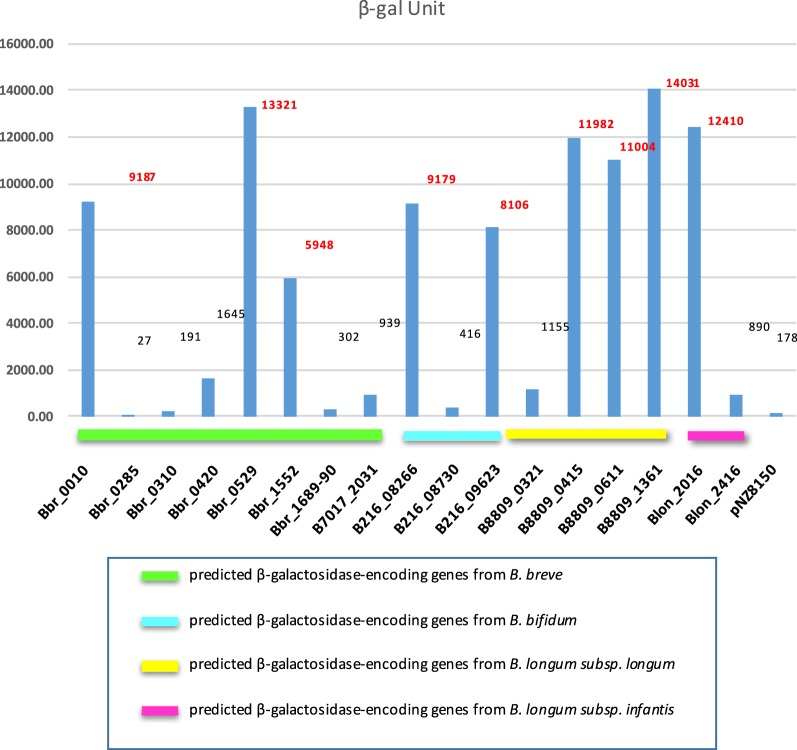
ONPG assay. Barplot showing the result of the ONPG assay performed on crude cell-free extracts of the 17 successfully cloned β-galactosidases. Crude cell-free extracts showing relative high level of enzymatic activity are indicated in red

In order to determine if the products of the 17 cloned genes were active on substrates other than or in addition to X-gal, β-galactosidase assays were conducted using crude cell extracts obtained from individual, nisin-induced cultures of *L. lactis* in which a predicted β-galactosidase-encoding gene had been cloned (Additional file 1: Figure S2). Out of the 17 different crude cell extracts tested, nine were shown to exhibit high activity against the artificial substrate ONPG (between 5000 and 14,000 Miller units) (Fig. 4). In addition, five crude cell-free extracts were demonstrated to display a relative low level of enzymatic activity (between 400 and 2000 Miller units) to cleave this ONPG substrate, while the remaining three crude cell-free extracts (corresponding to the expressed proteins specified by Bbr_0285, Bbr_0310 and Bbr_1689/1690) failed to show hydrolytic activity against this substrate (baseline set at 400 Miller units) (Fig. 4). It is worth mentioning that Bbr_0285 and Bbr_0310 also did not form blue colonies on plates, thus providing further confirmation of their presumed inactivity as β-galactosidases (at least on the substrates tested, though lack of activity may also be due to lack of expression or protein misfolding).

### Protein overproduction, purification and hydrolysis assay

All cloned (putative) β-galactosidase-encoding genes were then utilized for protein overproduction and subsequent His6-tagged protein purification using the *L. lactis* nisin-inducible protein production system (see “Materials and methods”). In order to verify the success of these steps, individually expressed and purified proteins were visualised by SDS-PAGE analysis followed by staining with Coomassie Brilliant Blue (Additional file 1: Figure S2). In order to assess substrate specificity of each of the 17 cloned (predicted) bifidobacterial β-galactosidases, qualitative hydrolysis assays by HPAEC-PAD analysis were performed using a number of (β-)galactosidic linkage-containing carbohydrates. The (putative) β-galactosidases were first tested for hydrolytic activity towards lactose, lactulose and β-d-galactotriose (Galβ1-4Galβ1-4Gal) (Table 4). The obtained results demonstrate that the purified proteins exhibited varying ability to cleave these different sugars. In the reaction with lactose the best activity (i.e. complete or partial hydrolysis of the substrate observed within the first hour of reaction) was exhibited by the proteins corresponding to Bbr_0010, Bbr_0529 and Bbr_1552 (all from *B. breve*), B216_08266 (from *B. bifidum*), B8809_0415, B8809_0611 and B8809_1361 (all from *B. longum* subsp. *longum*), and Blon_2016 (from *B. longum* subsp*. infantis*) (Table 4). When the reaction was performed with lactulose the most active proteins were those corresponding to locus tags Bbr_0529, Bbr_1552, B216_08266, B8809_0415, B8809_1361, Blon_2016 and Blon_2416 (Table 4). When the hydrolysis reaction was performed with the substrate β-d-galactotriose (Galβ1-4Galβ1-4Gal), the proteins corresponding to locus tags Bbr_0420, Bbr_0529, B216_08266, B216_08730, B8809_0321, B8809_0415, Blon_2016 and Blon_2416 were shown to hydrolyse the substrate within the first hour of the applied reaction conditions (Table 4). Following these three tests it was decided to eliminate the proteins corresponding to locus tags Bbr_0285, Bbr_0310, Bbr_1689/90, B216_8730, B216_09623 and B7017_2031 from further analyses because they were shown to be unable to hydrolyse any of the sugars tested. Notably, among these negatives are Bbr_0285 and Bbr_0310, which also did not show activity towards X-gal or ONPG substrates (Table 4; Fig. 4). In addition Bbr_1689/90, B216_09623 and B7017_2031 showed discordant activity towards hydrolytic activity towards X-gal, ONPG and lactose substrates. For this reason we decided to test their hydrolytic activity also on crude cell extract (which represents an unpurified enzyme; our purification method could have caused inactivation of the enzyme). Absence of protein expression was not considered to be the reason for the lack of activity of these enzymes because all these proteins were successfully overproduced and purified (Additional file 1: Figure S2). Based on the results of the hydrolysis assay on crude cell extracts, B216_09623 and B7017_2031 were shown to be capable of lactose hydrolysis, thus suggesting that the protein purification may have negatively affected their activity. Hydrolysis assays employing the remaining eleven β-galactosidases were then performed utilizing commercially available β-galactosyl-containing structures (including HMOs and GOS) as potential substrates. In the case of galactobiose (Galβ1-6Gal), clear hydrolysis activity was obtained when the reaction was performed with the proteins corresponding to locus tags Bbr_0529, Bbr_1552, B216_08266, B8809_0415 and Blon_2016. When the substrate galactotriose Galα1-3Galβ1-4Gal was used, no activity was detected within 24 h for any of the proteins tested, whereas four proteins (corresponding to Bbr_0420, Bbr_1552, B8809_0321, Blon_2416) were shown to be able to cleave galactobiose Galβ1-4Gal (Table 5). In order to further characterize the identified predicted β-galactosidases, their ability to hydrolyse the following carbohydrates, representing a selection of (β-linked galactose-containing) HMOs: lacto-*N*-tetraose (LNT), lacto-*N*-neotetraose (LNnT), 2′-fucosyllactose (2′-FL) and 3-fucosyllactose (3-FL) (Table 5). In the reaction with LNT the highest activity (positive within the first hour of reaction) was exhibited by the proteins corresponding to locus tags Bbr_0529 and Bbr_1552 (from *B. breve*), B216_08266 (from *B. bifidum*), B8809_0415, (from *B. longum* subsp. *longum*), and Blon_2016 (from *B. longum* subsp. *infantis*) (Table 5). When the reaction was performed with LNnT as a substrate, the most active proteins were those corresponding to locus tags Bbr_0010, Bbr_0529, Bbr_1552, B216_08266, B8809_0415, B8809_0611, B8809_1361 and Blon_2016 (Table 5). When the hydrolysis reaction was performed with the substrate 2′-FL, it was found that none of the proteins is active on this substrate (Table 5). The last sugar tested was 3-FL, and in this case the most active proteins were Bbr_0420, Bbr_0529, B216_08266, B8809_0321, B8809_0415, B8809_0611, B8809_1361 and Blon_2016 (Table 5).

**Table 5:**
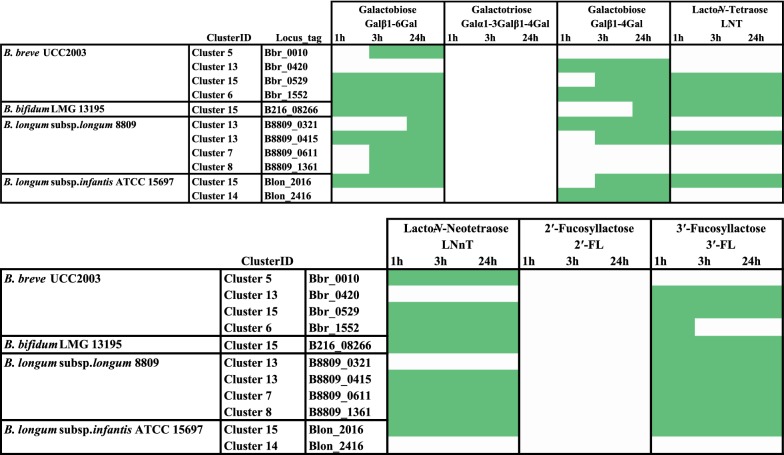
Hydrolysis performance of the 11 selected β-galactosidases tested on galactobiose, galactotriose, LNT, LNnT, 2′-FL and 3-FL

Key to colour coding: Substrate hydrolysis observed (green), no hydrolysis (white)

## Discussion

In the current work we investigated β-galactosidase enzymes belonging to four bifidobacterial species *B. breve*, *B. bifidum*, *B. longum* subsp. *longum* and *B. longum* subsp. *infantis*, all of which are commonly found in healthy, breast-fed infants. Our analysis clearly shows that multiple distinct β-galactosidases are encoded by these infant-derived bifidobacterial species (Additional file 1: Table S1) with a partial overlap between their hydrolytic activities (Tables 4 and 5). Notably, for some of the identified β-galactosidases the involvement in utilization of galacto-oligosaccharides and/or host-derived glycans has previously been reported (Additional file 1: Table S1). Of note, some of the clusters from our comparative analysis appeared to be species specific, suggesting that certain β-galactosidase encoding genes are only found within certain species. For example the enzymes belonging to Clusters 1, 2, 3, 6 and 9 contain a wide range of *B. bifidum* β-galactosidases which represent homologs of BbgIII (NCIMB 41171), BbgI (NCIMB 41171), BbgIV (NCIMB 41171), BIF2 (DSM 2015) and BIF1 (DSM 20215), respectively. Notably, these enzymes have previously been reported to be capable of GOS synthesis (Additional file 1: Table S1) (Goulas et al. 2007; Miwa et al. 2010; Moller et al. 2001).

Our comparative analysis was used as a guide to select 20 candidates to be used for further assessment of β-galactosidase activity towards a number of substrates including a range of selected β-galactosyl-containing structures. Cloning efforts of the selected genes resulted in the successful overexpression and purification of 17 presumed β-galactosidases, of which hydrolytic activity was further assessed on a variety of substrates. The obtained findings revealed that the majority of the expressed enzymes indeed exhibit β-galactosidase activity. However, these β-galactosidases clearly differ from each other in their substrate specificity, since some of the enzymes are active on nearly all tested substrates, while others appear to be quite specific and merely hydrolyse one or two substrates.

Of note, the enzymes that are capable of hydrolysing the broadest range of selected substrates are members of Cluster 15 and are also widespread across the four (sub)species (i.e. Bbr_0529, B216_08266, B8809_0415 and Blon_2016), suggesting that these genes encode conserved β-galactosidase activities. Besides, enzymes of Cluster 15 appear to be homologous to the previously identified BbgII (from B*. bifidum* NCIMB 41171), which is capable of both GOS hydrolysis and synthesis (Goulas et al. 2007; Miwa et al. 2010; Moller et al. 2001) (Fig. 1). Notably, some of the β-galactosidases represented by this cluster have previously been shown to be involved in the utilization of either GOS and HMOs (Additional file 1: Table S1). In particular, it has been shown that Blon_2016 from *B. longum* subsp. *infantis* ATCC 15697 represents a β-galactosidase possessing hydrolytic activity towards LNT and LNnT, as well as GOS (Garrido et al. 2013), which is indeed confirmed by our observations (Tables 4 and 5).

Another member of Cluster 15 is the β-galactosidase encoded by Bbr_0529, which is required for the utilization of GOS and certain HMOs by *B. breve* UCC2003 (James et al. 2016; O’Connell Motherway et al. 2013), being consistent with our observed hydrolytic activities towards the majority of tested substrates including galactobiose, galactotriose, but also the central moieties of type I and type II HMOs (Tables 3, 4). As type I chains represent the most abundant HMO core structure in human milk (Urashima et al. 2012), the prevalence of Cluster 15 members across all four infant-derived bifidobacterial (sub)species (Fig. 3b) and observed ability to cleave LNT (but also LNnT) (Table 4) supports their relevance to bifidobacteria in obtaining access to substrates derived from a human milk-based diet.

Surprisingly, one of the β-galactosidases whose product was shown to be incapable of hydrolysing lactose or lactulose is Bbr_0420, which was instead shown to be active towards d-galactotriose Galβ1-4Galβ1-4Gal (Table 4). Previous studies established that Bbr_0420, a β-galactosidase which is upregulated when *B. breve* UCC2003 is grown on purified GOS (O’Connell Motherway et al. 2013), seems to be dedicated to the hydrolysis of d-galactotriose, which in turn is produced from the extracellular degradation of potato galactan in this strain (O’Connell Motherway et al. 2011). The transcription of this gene is, however, not induced when *B. breve* UCC2003 is grown on lactose and certain HMOs (e.g. LNT or LNnT) (James et al. 2016). Bbr_0420 from *B. breve* UCC2003 and its homologue B8809_0321 from *B. longum* subsp. *longum* NCIMB 8809 (both belonging to Cluster 13) were shown here to hydrolyse galactobiose (Galβ1-4Gal) and galactotriose (Galβ1-4Gal-β1-4Gal), but incapable of hydrolysing either LNT or LNnT. This suggests that they represent β-galactosidases with a narrow substrate specificity, which is directed towards β1,4 galactosidic links (Tables 4, 5). Interestingly, a recent study has associated a homolog corresponding to this β-galactosidase and located within the galactan cluster of *B. longum* AH1206 (BL1206_0411) with the persistence of this strain in the GIT tract (Maldonado-Gomez et al. 2016). This finding represents another indication of the role played by β-galactosidases and their dietary galactose-containing substrates in promoting bifidobacterial colonization.

Notably, purified Bbr_0010 from *B. breve* UCC2003 has previously been reported to hydrolyze lactose and LNnT (James et al. 2016), while Bbr_1552 represents a β-galactosidase with a broader substrate specificity, being involved in the hydrolysis of GOS, as well as lactose, LNT and LnNT in this strain (James et al. 2016; O’Connell Motherway et al. 2013), which is also consistent with our observations. Interestingly, a previous study employing a Tn5-based random mutagenesis system in *B. breve* UCC2003 identified Bbr_0010 as the main β-galactosidase responsible for lactose utilization in this strain (Ruiz et al. 2013). Based on our analysis this enzyme does indeed hydrolyse lactose (as well as LNnT) and the corresponding gene is conserved across *B. breve* (as Cluster 5 members).

Two interesting cases are constituted by B8809_0611 (Cluster 7) and B8809_1361 (Cluster 8) from *B. longum* subsp. *longum* NCIMB 8809 as these two β-galactosidases show similar hydrolytic profile and are particularly active towards LNnT (type II chain) despite being member of two different clusters (Tables 4 and 5). Cluster 7 member Blon_2334 from *B. longum* subsp. *infantis* ATCC 15697 has been described as a β-galactosidase responsible for degradation of lactose and type II HMOs, but not type I HMOs (Yoshida et al. 2012), being consistent with what we observed for B8809_0611. In contrast, B8809_1361, which shows a hydrolytic profile similar to Blon_2334 and B8809_0611, belongs to a different cluster (Cluster 8). Finally, B8809_1361 is only present in certain strains of *B. longum* subsp. *longum*, and perhaps constitutes an auxiliary β-galactosidase responsible for degradation of lactose and type II HMOs in this subspecies (Additional file 1: Table S1).

In conclusion, the information collected in this study highlights the importance of GHs in bifidobacterial saccharolytic metabolism, in particular β-galactosidases which are involved in the utilization of a range of substrates such as lactose, HMOs, and GOS, found as part of the milk-based infant diet. The qualitative assay presented in this study provides a clear insight on the diversity of β-galactosidases in terms of substrate specificity. In fact, some appear to be more specialized towards milk-based substrates, while others are specific for plant-derived substrates. Therefore, the findings presented here constitute a solid foundation for future studies on bifidobacterial β-galactosidases and further investigation on the role of milk-derived substrates in establishing bifidobacterial predominance in the infant GIT.

## Additional file

**Additional file 1: Table S1.** List of bifidobacterial genes used for comparative purposes. **Figure S1.** *In silico* analysis and functional characterization of β-galactosidases. **Figure S2.** SDS-PAGE of purified proteins.
