# Supplementary material for: Characterization of GH2 and GH42 β-galactosidases derived from bifidobacterial infant isolates
Source: AMB Express. 2019 Jan 19;9:9. doi: 10.1186/s13568-019-0735-3 (PMC6339635; doi:10.1186/s13568-019-0735-3)
Supplement: Supplementary file 1 — Additional file 1: Table S1. List of bifidobacterial genes used for comparative purposes. Figure S1. In silico analysis and functional characterization of β-galactosidases. Figure S2. SDS-PAGE of purified proteins. [file 13568_2019_735_MOESM1_ESM.pdf]

SUPPLEMENTARY MATERIAL

Journal name: AMB Express

Title: Characterization of GH2 and GH42 β-galactosidases derived from bifidobacterial infant isolates.

Valentina Ambrogi[1,2], Francesca Bottacini[1], Joyce O'Sullivan[1], Mary O'Connell Motherway[1], Cao Linqiu[3], Barry Schoemaker[3], Margriet Schoterman[3] and Douwe van Sinderen[1,2]*.

[1]APC Microbiome Ireland, University College Cork, Western Road, Cork, Ireland

[2]School of Microbiology, National University of Ireland, Western Road, Cork, Ireland

[3] FrieslandCampina, Amersfoort, the Netherlands

* Corresponding author.

Corresponding author email address: d.vansinderen@ucc.ie

Running title: Bifidobacterial β-galactosidases

Table S1. List of bifidobacterial genes used for comparative purposes.

ClusterID	Locus_tag	Species	Strain	Gene size (aa)	Accession	GH family	Reference
**Cluster1**	BbgIII	*B. bifidum*	JCM 1254	1935	BAI94821	GH2	Miwa et al. 2010
**Cluster1**	BIF3	*B. bifidum*	DSM 20215	1752	CAC14566	GH2	Moller et al. 2001
**Cluster1**	BBBF_0474	*B. bifidum*	DSM 20456	1935	BAQ97681	GH2	
**Cluster1**	B216_06500	*B. bifidum*	LMG 13195	1803	EKE50382	GH2	
**Cluster1**	BBB_0459	*B. bifidum*	BGN4	1935	AFL04054	GH2	
**Cluster1**	BBNG_00396 (BbgIII)	*B. bifidum*	NCIMB 41171	1891	EFR49849	GH2	Goulas et al. 2007
**Cluster1**	BBPR_0482	*B. bifidum*	PRL2010	1935	ADP35589	GH2	Turroni et al. 2010
**Cluster1**	BBIF_0507	*B. bifidum*	S17	1935	ADO52712	GH2	
**Cluster2**	BbgI	*B. bifidum*	JCM 1254	1291	BAI94825	GH2	Miwa et al. 2010
**Cluster2**	BBBF_0159	*B. bifidum*	DSM 20456	1292	BAQ97366	GH2	
**Cluster2**	BBB_0145	*B. bifidum*	BGN4	1291	AFL03741	GH2	
**Cluster2**	B217_01231	*B. bifidum*	IPLA20015	1292	EKF16574	GH2	
**Cluster2**	BIFI_0651	*B. bifidum*	LMG 11041	1292	KFI42266	GH2	
**Cluster2**	BBNG_00071 (BbgI)	*B. bifidum*	NCIMB 41171	1291	EFR49525	GH2	Goulas et al. 2007
**Cluster2**	B216_09411	*B. bifidum*	LMG 13195	1292	EKE49818	GH2	
**Cluster2**	BBPR_0150	*B. bifidum*	PRL2010	1292	ADP35284	GH2	Turroni et al. 2010
**Cluster2**	BBIF_0188	*B. bifidum*	S17	1292	ADO52393	GH2	
**Cluster3**	BbgIV	*B. bifidum*	JCM 1254	1052	BAI94821	GH2	Miwa et al. 2010
**Cluster3**	BBBF_1456	*B. bifidum*	DSM 20456	1052	BAQ98663	GH2	
**Cluster3**	BBB_1439	*B. bifidum*	BGN4	1052	AFL05030	GH2	
**Cluster3**	B217_02490	*B. bifidum*	IPLA20015	1052	EKF16333	GH2	
**Cluster3**	BIFI_0119	*B. bifidum*	LMG 11041	1052	KFI43782	GH2	
**Cluster3**	B216_09623	*B. bifidum*	LMG 13195	1052	EKE49770	GH2	
**Cluster3**	BBNG_01638 (BbgIV)	*B. bifidum*	NCIMB 41171	1052	EFR51090	GH2	Goulas et al. 2007
**Cluster3**	BBPR_1460	*B. bifidum*	PRL2010	1052	ADP36493	GH2	Turroni et al. 2010

**Cluster3**	BBIF_1406	*B. bifidum*	S17	1053	ADO53611	GH2	
**Cluster6**	BIF2	*B. bifidum*	DSM 20215	1044	CAC14565	GH2	Moller et al. 2001
**Cluster9**	BIF1	*B. bifidum*	DSM 20215	1020	CAB75342	GH2	Moller et al. 2001
**Cluster10**	B217_07459	*B. bifidum*	IPLA20015	987	EKF15369	GH2	
**Cluster12**	BbgV	*B. bifidum*	JCM 1254	740	BAI94828	GH42	Miwa et al. 2010
**Cluster12**	BBBF_0172	*B. bifidum*	DSM 20456	739	BAQ97379	GH42	
**Cluster12**	BBB_0160	*B. bifidum*	BGN4	726	AFL03756	GH42	
**Cluster12**	BIFI_0664	*B. bifidum*	LMG 11041	726	KFI42279	GH42	
**Cluster12**	B216_08730	*B. bifidum*	LMG 13195	714	EKE49942	GH42	
**Cluster12**	BBNG_00089	*B. bifidum*	NCIMB 41171	726	EFR49543	GH42	
**Cluster12**	BBPR_0164	*B. bifidum*	PRL2010	726	ADP35297	GH42	
**Cluster15**	BbgII	*B. bifidum*	JCM 1254	689	BAI94826	GH42	Miwa et al. 2010
**Cluster15**	BBBF_1344	*B. bifidum*	DSM 20456	693	BAQ98551	GH42	
**Cluster15**	BBB_1337	*B. bifidum*	BGN4	689	AFL04929	GH42	
**Cluster15**	BBPR_1355	*B. bifidum*	PRL2010	690	ADP36405	GH42	Turroni et al. 2010
**Cluster15**	BIFI_0010	*B. bifidum*	LMG 11041	689	KFI43682	GH42	
**Cluster15**	B216_08266	*B. bifidum*	LMG 13195	693	EKE50024	GH42	
**Cluster15**	BBNG_01731 (BbgII)	*B. bifidum*	NCIMB 41171	689	EFR51183	GH42	Goulas et al. 2007
**Cluster15**	BBIF_1313	*B. bifidum*	S17	689	ADO53518	GH42	
**Cluster16**	B217_02337	*B. bifidum*	IPLA20015	609	EKF16372	GH42	
**Cluster3**	B2L_0246	*B. breve*	2L	1163	EWH42012	GH2	
**Cluster3**	B7019_1720	*B. breve*	JCM 7019	1052	AHJ19889	GH2	
**Cluster4**	Bbr_1689-90	*B. breve*	UCC2003	157+1054	ABE96355-56	GH2	
**Cluster4**	B31L_1766-67	*B. breve*	31L	157+1054	EWH39417-18	GH2	
**Cluster4**	B2258_1707-08	*B. breve*	NCFB 2258	157+1054	AHJ21881-82	GH2	
**Cluster4**	BS27_1679-80	*B. breve*	S27	157+1054	AHJ25446-47	GH2	
**Cluster5**	Bbr_0010	*B. breve*	UCC2003	1043	ABE94727	GH2	Ruiz et al. 2013; James et al. 2016
**Cluster5**	B2L_0837	*B. breve*	2L	1043	EWH42557	GH2	
**Cluster5**	B12L_0010	*B. breve*	12L	1043	AHJ14821	GH2	

**Cluster5**	B31L_0439	*B. breve*	31L	1043	EWH41715	GH2	
**Cluster5**	B7017_0010	*B. breve*	JCM 7017	1043	AHJ16586	GH2	
**Cluster5**	B7019_0010	*B. breve*	JCM 7019	1043	AHJ18356	GH2	
**Cluster5**	HMPREF1587_01718	*B. breve*	JCP 7499	1051	ERI86567	GH2	
**Cluster5**	B2258_0010	*B. breve*	NCFB 2258	1043	AHJ20271	GH2	
**Cluster5**	BS27_0010	*B. breve*	S27	1043	AHJ23926	GH2	
**Cluster6**	Bbr_1552	*B. breve*	UCC2003	1045	ABE96223	GH2	O' Connell Motherway et al. 2012; James et al. 2016
**Cluster6**	B12L_1479	*B. breve*	12L	1045	AHJ16208	GH2	
**Cluster6**	B31L_1616	*B. breve*	31L	1045	EWH39283	GH2	
**Cluster6**	B7017_1745	*B. breve*	JCM 7017	1045	AHJ17983	GH2	
**Cluster6**	HMPREF1587_01118	*B. breve*	JCP 7499	1045	ERI87315	GH2	
**Cluster6**	B2258_1567	*B. breve*	NCFB 2258	1045	AHJ21745	GH2	
**Cluster6**	BS27_1534	*B. breve*	S27	1044	AHJ25307	GH2	
**Cluster13**	B2L_1156	*B. breve*	2L	710	EWH41313	GH42	
**Cluster13**	B12L_0358	*B. breve*	12L	710	AHJ15148	GH42	
**Cluster13**	B31L_0023	*B. breve*	31L	710	EWH41352	GH42	
**Cluster13**	B7017_0374	*B. breve*	JCM 7017	710	AHJ16917	GH42	
**Cluster13**	B7019_0383	*B. breve*	JCM 7019	710	AHJ18699	GH42	
**Cluster13**	HMPREF1587_00395	*B. breve*	JCP 7499	710	ERI87956	GH42	
**Cluster13**	B2258_0392	*B. breve*	NCFB 2258	710	AHJ20623	GH42	
**Cluster13**	Bbr_0420	*B. breve*	UCC2003	710	ABE95118	GH42	O' Connell Motherway et al. 2012
**Cluster13**	BS27_0422	*B. breve*	S27	710	AHJ24299	GH42	
**Cluster14**	B7017_2031	*B. breve*	JCM 7017	701	AHJ18248	GH42	
**Cluster15**	B2L_1017	*B. breve*	2L	691	EWH41185	GH42	
**Cluster15**	B12L_0447	*B. breve*	12L	691	AHJ15235	GH42	
**Cluster15**	B31L_1298	*B. breve*	31L	691	EWH41023	GH42	
**Cluster15**	B7017_0482	*B. breve*	JCM 7017	691	AHJ17019	GH42	

**Cluster15**	B7019_0489	*B. breve*	JCM 7019	690	AHJ18798	GH42	
**Cluster15**	HMPREF1587_00540	*B. breve*	JCP 7499	691	ERI87893	GH42	
**Cluster15**	B2258_0481	*B. breve*	NCFB 2258	691	AHJ20707	GH42	
**Cluster15**	BS27_0520	*B. breve*	S27	690	AHJ24385	GH42	
**Cluster15**	Bbr_0529	*B. breve*	UCC2003	691	ABE95226	GH42	O' Connell Motherway et al. 2012; James et al 2016
**Cluster17**	Bbr_0285	*B. breve*	UCC2003	606	ABE94991	GH2	
**Cluster18**	B7019_0853	*B. breve*	JCM 7019	586	AHJ19136	GH2	
**Cluster18**	Bbr_0310	*B. breve*	UCC2003	586	ABE95012	GH2	
**Cluster7**	B8809_0611	*B. longum* subsp. *longum*	NCIMB 8809	1023	ALO72284	GH2	
**Cluster7**	EK13BL_04235	*B. longum* subsp. *longum*	EK13	1023	KEY32734	GH2	
**Cluster7**	BIL_12070	*B. longum* subsp. *longum*	F8	1023	CBK70710	GH2	
**Cluster7**	BLJ_0749	*B. longum* subsp. *longum*	JDM301	1023	ADH00219	GH2	
**Cluster7**	BLNIAS_01829	*B. longum* subsp. *longum*	KACC 91563	1023	AEI97790	GH2	
**Cluster7**	NL89_01385	*B. longum* subsp. *longum*	VMKB44	1023	KHD96107	GH2	
**Cluster8**	B8809_1361	*B. longum* subsp. *longum*	NCIMB 8809	1063	ALO73032	GH2	
**Cluster8**	HMPREF0175_1109	*B. longum* subsp. *longum*	ATCC 55813	1064	EEI80881	GH2	
**Cluster8**	BBMN68_1812	*B. longum* subsp. *longum*	BBMN68	1063	ADQ02870	GH2	
**Cluster8**	BLLJ_1486	*B. longum* subsp. *longum*	JCM 1217	1063	BAJ67153	GH2	
**Cluster8**	BLSL_1472	*B. longum* subsp. *longum*	LMG 13197	1063	KFI62448	GH2	
**Cluster11**	BLJ_1934	*B. longum* subsp. *longum*	JDM301	788	ADH01357	GH42	
**Cluster13**	HMPREF0175_0277	*B. longum* subsp. *longum*	ATCC 55813	719	EEI81809	GH42	
**Cluster13**	BLLJ_0352	*B. longum* subsp. *longum*	JCM 1217	719	BAJ66022	GH42	
**Cluster13**	NL89_05225	*B. longum* subsp. *longum*	VMKB44	719	KHD95104	GH42	
**Cluster13**	B8809_0321	*B. longum* subsp. *longum*	NCIMB 8809	710	ALO71994	GH42	
**Cluster13**	BN57_524	*B. longum* subsp. *longum*	CECT 7347	710	CCK34389	GH42	
**Cluster13**	BBMN68_1021	*B. longum* subsp. *longum*	BBMN68	710	ADQ01982	GH42	
**Cluster13**	EK13BL_10545	*B. longum* subsp. *longum*	EK13	710	KEY33117	GH42	
**Cluster13**	BIL_15140	*B. longum* subsp. *longum*	F8	710	CBK70970	GH42	

**Cluster13**	BLJ_0417	*B. longum* subsp. *longum*	JDM301	710	ADG99893	GH42	
**Cluster13**	BLNIAS_02287	*B. longum* subsp. *longum*	KACC 91563	710	AEI98104	GH42	
**Cluster13**	BLSL_0343	*B. longum* subsp. *longum*	LMG 13197	675	KFI64946	GH42	
**Cluster15**	B8809_0415	*B. longum* subsp. *longum*	NCIMB 8809	691	ALO72088	GH42	
**Cluster15**	HMPREF0175_0769	*B. longum* subsp. *longum*	ATCC 55813	691	EEI81198	GH42	
**Cluster15**	BN57_621	*B. longum* subsp. *longum*	CECT 7347	691	CCK34482	GH42	
**Cluster15**	BBMN68_930	*B. longum* subsp. *longum*	BBMN68	691	ADQ01884	GH42	
**Cluster15**	EK13BL_02710	*B. longum* subsp. *longum*	EK13	691	KEY32452	GH42	
**Cluster15**	BIL_14030	*B. longum* subsp. *longum*	F8	691	CBK70878	GH42	
**Cluster15**	BLLJ_0443	*B. longum* subsp. *longum*	JCM 1217	691	BAJ66112	GH42	
**Cluster15**	BLJ_0519	*B. longum* subsp. *longum*	JDM301	691	ADG99995	GH42	
**Cluster15**	BLSL_0435	*B. longum* subsp. *longum*	LMG 13197	691	KFI65038	GH42	
**Cluster15**	NL89_00375	*B. longum* subsp. *longum*	VMKB44	691	KHD95934	GH42	
**Cluster4**	BLIF_0281-82	*B. longum* subsp. *infantis*	157F	157+1054	BAJ70428-29	GH2	
**Cluster7**	Blon_2334	*B. longum* subsp. *infantis*	ATCC 15697	1023	ACJ53392	GH2	Garrido et al. 2013; Sela et al 2008
**Cluster7**	EK3BL_00585	*B. longum* subsp. *infantis*	EK3	1023	KEY30718	GH2	
**Cluster7**	BLIG_00914	*B. longum* subsp. *infantis*	CCUG 52486	1023	EEQ54963	GH2	
**Cluster7**	BLIF_0659	*B. longum* subsp. *infantis*	157F	1023	BAJ70802	GH2	
**Cluster12**	Blon_2123	*B. longum* subsp. *infantis*	ATCC 15697	720	ACJ53184	GH42	Garrido et al. 2013
**Cluster12**	EK3BL_09165	*B. longum* subsp. *infantis*	EK3	720	KEY26226	GH42	
**Cluster13**	BLIF_0351	*B. longum* subsp. *infantis*	157F	710	BAJ70496	GH42	
**Cluster13**	BLIG_00257	*B. longum* subsp. *infantis*	CCUG 52486	710	EEQ54308	GH42	
**Cluster14**	Blon_2416	*B. longum* subsp. *infantis*	ATCC 15697	706	ACJ53470	GH42	Garrido et al. 2013
**Cluster15**	Blon_2016	*B. longum* subsp. *infantis*	ATCC 15697	691	ACJ53083	GH42	Garrido et al. 2013; Sela et al 2008
**Cluster15**	BLIF_0460	*B. longum* subsp. *infantis*	157F	691	BAJ70604	GH42	
**Cluster15**	BLIG_00161	*B. longum* subsp. *infantis*	CCUG 52486	691	EEQ54212	GH42	
**Cluster15**	EK3BL_06145	*B. longum* subsp. *infantis*	EK3	691	KEY27369	GH42	
**Cluster17**	Blon_0268	*B. longum* subsp. *infantis*	ATCC 15697	606	ACJ51396	GH42	Garrido et al. 2013

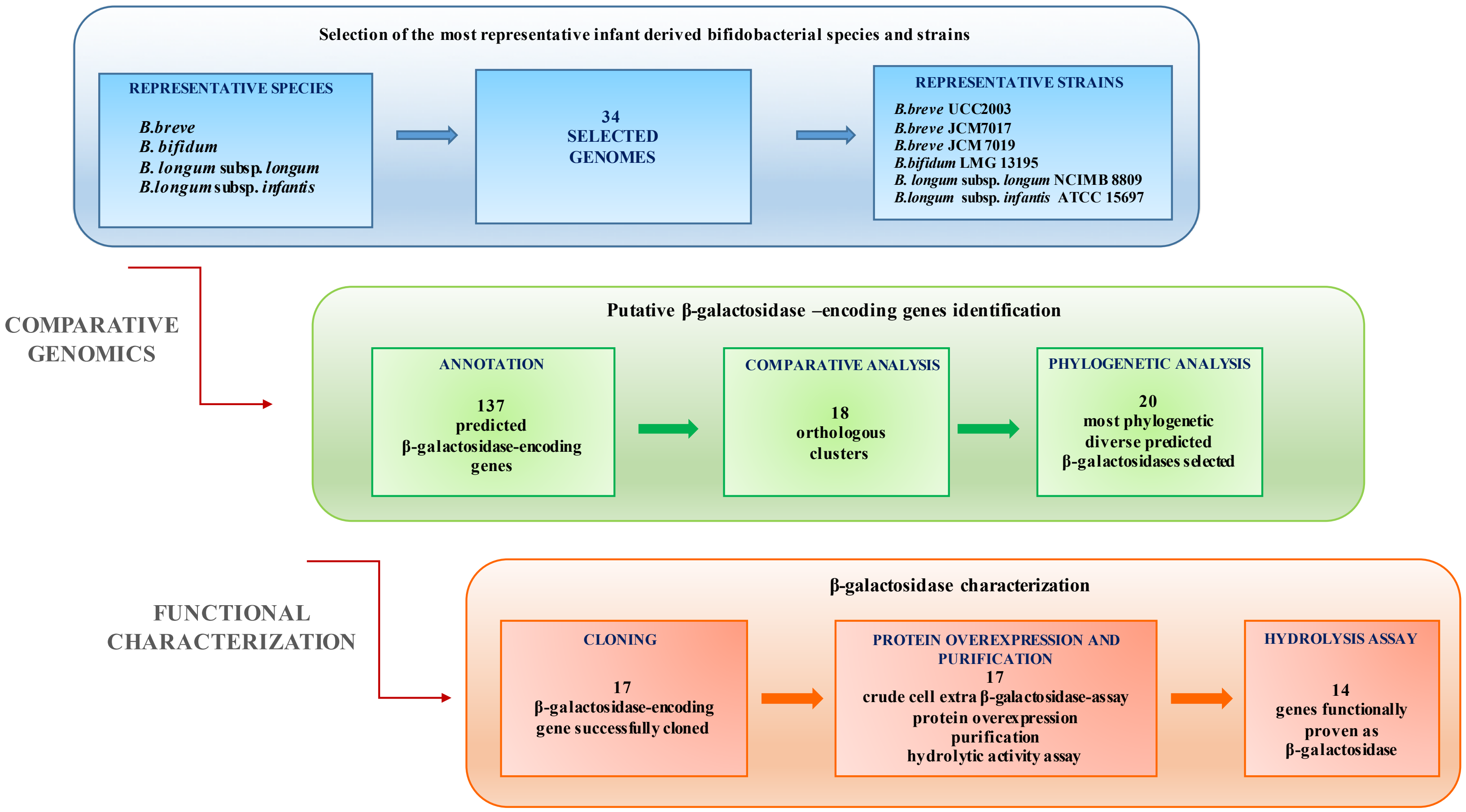

Figure S1. ***In silico*** **analysis and functional characterization of β-galactosidases.**

Schematic representation of the single steps necessary to perform genome analysis, comparative genomics and functional characterization of bifidobacterial β-galactosidase encoding genes.

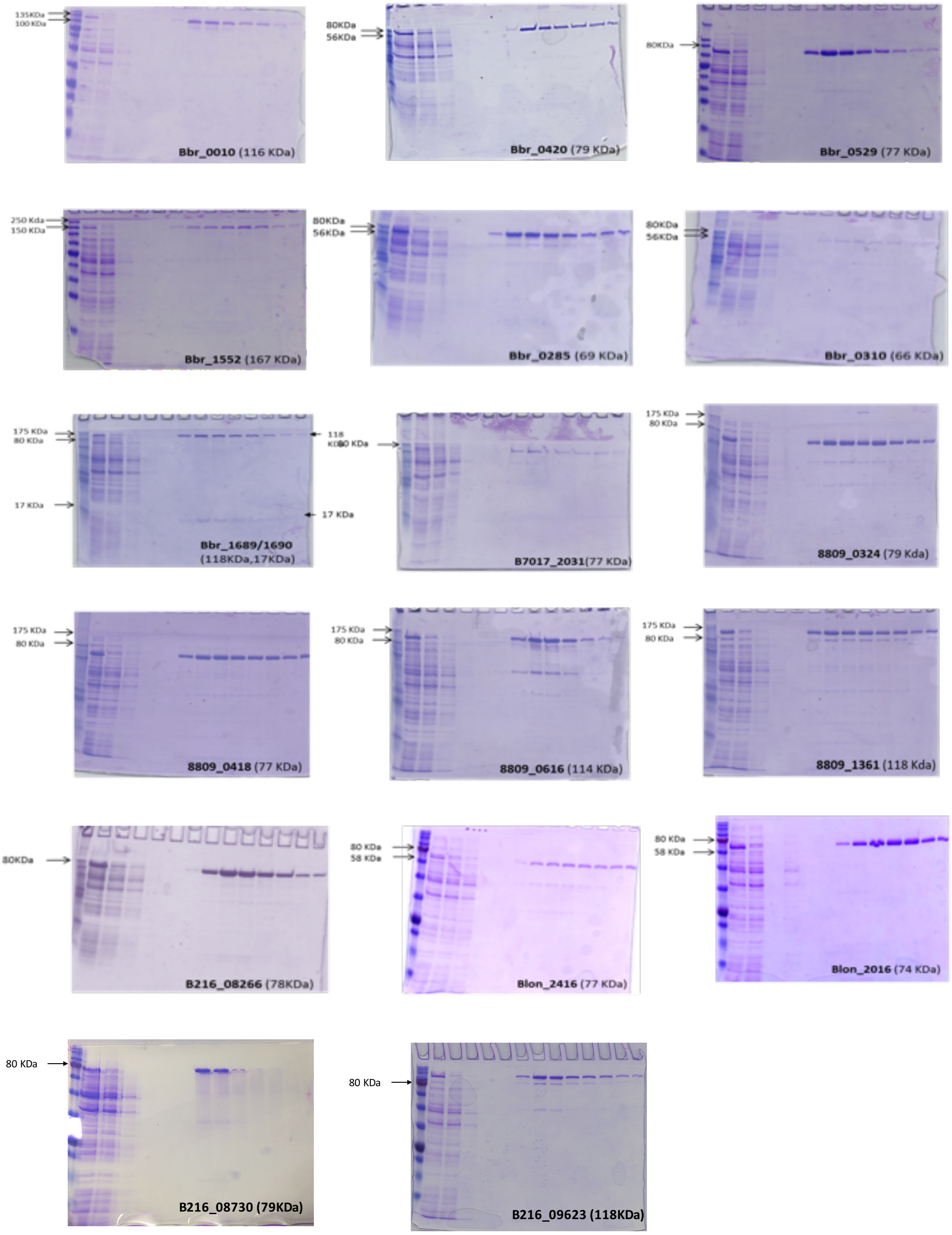

Figure S2. SDS-PAGE of purified proteins.

Purified bifidobacterial β-galactosidases visualised by SDS-polyacrylamide gel electrophoreses (SDS_PAGE). The expected protein size is indicated beside each gene name, while black arrows highlight the range of the obtained protein size.
